# A supercritical oil extract of *Schisandra chinensis* seeds ameliorates Huntington’s disease-like symptoms and neuropathology: the potential role of anti-oxidant and anti-inflammatory effects

**DOI:** 10.3389/fphar.2024.1471024

**Published:** 2024-12-23

**Authors:** Hyo-Sung Jo, Youn-Woo Lee, So-Ri Son, Dae Sik Jang, Tae Woo Kwon, Yujeong Ha, Sang-Kwan Moon, Min Soo Kim, Ik-Hyun Cho

**Affiliations:** ^1^ Department of Convergence Medical Science, College of Korean Medicine, Kyung Hee University, Seoul, Republic of Korea; ^2^ Department of Science in Korean Medicine, Graduate School, Kyung Hee University, Seoul, Republic of Korea; ^3^ School of Chemical and Biological Engineering, Institute of Chemical Processes, Seoul National University, Seoul, Republic of Korea; ^4^ Department of Pharmaceutical Science, College of Pharmacy, Kyung Hee University, Seoul, Republic of Korea; ^5^ Department of Clinical Korean Medicine, Graduate School, Kyung Hee University, Seoul, Republic of Korea; ^6^ Brain Science Institute, Korea Institute of Science and Technology (KIST), Seoul, Republic of Korea; ^7^ KHU-KIST Department of Converging Science and Technology, Kyung Hee University, Seoul, Republic of Korea; ^8^ Institute of Convergence Korean Medicine, College of Korean Medicine, Kyung Hee University, Seoul, Republic of Korea

**Keywords:** seed oil of Schisandra chinensis, Huntington’s disease-like symptoms, anti-inflammation, antioxidant, neuroprotection

## Abstract

**Background:**

Huntington disease (HD), a neurodegenerative autosomal dominant disorder, is characterized by involuntary choreatic movements with cognitive and behavioral disturbances. Up to now, no therapeutic strategies are available to completely ameliorate the progression of HD. *Schisandra chinensis* has various pharmacologic effects such as antioxidant and anti-inflammatory activities. However, the neuroprotective value of seed oil of *S. chinensis* (SOSC) has not been elucidated yet. The purpose of this study was to determine neuroprotective effects of SOSC by supercritical fluid extraction against 3-nitropropionic acid (3-NPA)-induced HD-like symptoms and neuropathology in an experimental mouse model.

**Methods:**

SOSC (75, 150, and 300 mg/kg/day) was orally pre-administration once daily at 1 hour before 3-NPA intoxication.

**Results:**

SOSC ameliorated movement dysfunction and lethality following 3-NPA intoxication in connection with reduction of lesion area, neurodegeneration/apoptosis, microglial migration/activation, and mRNA expression of pro-inflammatory cytokines/enzymes in the striatum. SOSC inhibited the activation of nuclear factor-kappa B (NF-κB) and mitogen-activated protein kinase (MAPKs) pathways but stimulated nuclear factor erythroid 2-related factor 2 (Nrf2) in the striatum after 3-NPA intoxication. Schizandrin, a main component of SOSC, reduced protein expression levels of Iba-1 and p-NF-κB in 3-NPA-induced BV2 cells (murine microglia cell line). BV2 cell’s conditioned medium inhibited cleaved caspase-3 in 3-NPA-induced SH-SY5Y cells (a neuroblastoma cell line).

**Conclusion:**

SOSC might ameliorate movement dysfunction by inhibiting neuropathology through its anti-inflammatory and antioxidant activities in the striata of 3-NPA-intoxicated mice. These findings suggest that SOSC could serve as a promising therapeutic candidate for HD-like symptoms, providing a foundation for future treatment strategies targeting neuroinflammation and oxidative stress.

## 1 Introduction

Huntington’s disease (HD), also known as Huntington’s chorea, is an incurable neurodegenerative disease that is mostly inherited (Damiano et al., 2010; Ross and Tabrizi, 2011; Tabrizi et al., 2020). Neurodegeneration in HD is caused by the CAG repeat expansion of the huntingtin gene (*HTT*) encoding an abnormally long polyglutamine tract in the huntingtin protein within various brain areas such as the basal ganglia and cerebral cortex (Damiano et al., 2010; Ross and Tabrizi, 2011; Tabrizi et al., 2020). Abnormal aggregation of mutant HTT (mHTT)-protein can lead to multiple pathological elements, including neuronal toxicity, excitotoxicity, mitochondrial dysfunction, transcriptional dysfunction, alterations in axonal transport, and synaptic dysfunction (Damiano et al., 2010; Ross and Tabrizi, 2011; Tabrizi et al., 2020). HD typically leads to a combination of chorea, cognitive impairment, and psychiatric symptoms in patients (Damiano et al., 2010; Ross and Tabrizi, 2011; Tabrizi et al., 2020). Inflammation is frequently observed in HD patients before symptom onset. Neuroinflammation characterized by the presence of reactive microglia, astrocytes, and inflammatory factors within the brain is also detected early (Kabba et al., 2017; Tai et al., 2007). Nuclear factor kappa B (NF-κB) and mitogen-activated protein kinases (MAPKs) signaling pathways are pivotal transcription factors for microglial activation and cytokine production in neuroinflammation related to HD (El Kasmi et al., 2006; Kwon et al., 2017). Oxidative stress is also involved in the development and progression of HD (Singh et al., 2019; Teleanu et al., 2022). Various anti-inflammatory and antioxidant agents have beneficial effects in experimental models of HD and patients with HD (Atanasov et al., 2021; Gil-Mohapel et al., 2014; Tabrizi et al., 2020). Therefore, anti-inflammatory and antioxidant agents are considered as attractive therapeutic options for HD.

Few therapeutics have been clinically proven to be effective in targeting the pathological mechanisms underlying HD and improving its main symptoms (chorea and psychosis) (Huntington Study, 2006). Tetrabenazine (Xenazine^®^) is currently the only medication for treating HD. It was approved in 2000 in the EU and in 2008 in the US (Yero and Rey, 2008). Some newer antipsychotic agents (olanzapine and aripiprazole) might have adequate efficacy with more favorable adverse-effect profiles than older antipsychotic agents for treating chorea and psychosis (Dash and Mestre, 2020). Although some symptomatic treatments are available, these treatments might cause serious side effects, such as akathisia, depression, dizziness, and fatigue (Huntington Study, 2006). Unfortunately, the exact mechanism underlying neuronal death in HD has not been fully elucidated. Additionally, there is no disease-modifying treatment for HD. Therefore, there is an urgent need to develop neuroprotective drugs or other therapies (Tabrizi et al., 2020).

There is an increasing use of natural products, including medicinal plants, phytopharmaceuticals, nutraceuticals, vitamins, and nutritional supplements worldwide because of their safety and favorable efficacy in improving physical strength and/or treating diseases (Atanasov et al., 2021; Zhu et al., 2022). *Schisandra (S.) chinensis,* commonly known as *Omija* in Korean and *Wǔ wèi zi* in Chinese, meaning ‘five-flavor berry’, is a plant species that belongs to the genus *Schisandra* of the family Schisandraceae. *S. chinensis* has been used as a traditional Oriental medicine for two thousand years and is mainly distributed and cultivated in northeastern China, far-eastern Russia, Japan, and Korea (Kim et al., 2017; Nowak et al., 2019; Szopa et al., 2017). Recently, *S. chinensis* has attracted much attention due to its various pharmacologic effects, such as antioxidant and anti-inflammatory activities on different body systems including nervous and immune systems (Panossian and Wikman, 2008). *S. chinensis* has various active compounds including lignans, nortriterpenes, sesquiterpenes, and phenolic acids (Yang and Yuan, 2021). In recent years, mass production has become common due to advanced cultivation technology. Water and sugar extracts of fresh or dried *S. chinensis* are widely used in various foods such as tea, beverages, and health functional foods (Nowak et al., 2019). In this process, seeds of *S. chinensis* (SSC) are discarded without special use as by-products after primary processing such as water extraction and sugar extraction using *S. chinensis.* Seeds of *S. chinensis* have higher contents of lignans including schizandrin, soluble nitrogen-free extract, fatty acids including linoleic acid, and inorganic ions than other parts of *S. chinensis* such as peel, pulp, and so on (Lee et al., 2021). To effectively utilize *S. chinensis*, SSC must be fully identified and utilized for its functionality.

Supercritical fluid extraction (SFE) is a technique that extracts a specific component from a liquid or solid phase using the different solubility of substances in their supercritical or non-supercritical states (Uwineza and Waskiewicz, 2020). With increasing public interest in natural products, SFE is classified as one of the novel and standard extraction techniques for studying herbal, food, and agricultural samples (Uwineza and Waskiewicz, 2020). Recently, the chemical compositions of essential oil from *S. chinensis* obtained by SFE technique and its pharmacological activities, such as anti-inflammatory and antioxidant effects have been reported (Lee et al., 2021). Nevertheless, neuropharmacological role of the oil from *S. chinensis* itself or its seeds remains unclear.

As a metabolite of 3-nitropropanol, 3-nitropropionic acid (3-NPA) is a naturally occurring toxin that has been found in various fungal species, including Aspergillus flavus, Astragalus, and Arthrinium (Brouillet et al., 2005; Tunez et al., 2010). Systematically administration of 3-NPA into experimental rodent models can cause striatal toxicity, which closely mimics and reproduces behavioral (hyperkinetic and hypokinetic movements), histopathological, and neurochemical pathological features observed in HD (Brouillet et al., 2005; Tunez et al., 2010). Thus, 3-NPA has been used as an efficient chemical to induce HD-like symptoms and pathological features in animal models to study HD (Upadhayay et al., 2023).

The aim of the present study was to determine whether seed oil of *Schisandra chinensis* (SOSC) obtained by SFE could ameliorate HD-like symptoms and neuropathology through its antioxidant and anti-inflammatory mechanisms.

## 2 Methods and methods

### 2.1 Reagents

Rabbit anti-extracellular signal-regulated kinases 1/2 (ERK1/2; #9102), rabbit anti-interleukin-1 beta (IL-1β; #31202), rabbit anti-IL-6 (#12912), rabbit anti-phospho (p)-ERK1/2 (#9101), rabbit anti-c-Jun N-terminal kinases (JNK; #9258), rabbit anti-p-JNK (#4668), rabbit anti-NF-kappa-B inhibitor alpha (IκB-α; #4814), rabbit anti-p-IκB-α (#2859), rabbit anti-pro-caspase-3 (#9662), rabbit anti-cleaved caspase-3 (#9661), rabbit anti-p38 mitogen-activated protein kinase (MAPK; #9212), mouse anti-p-p38 MAPK (#9216), and rabbit anti-transforming growth factor-beta (TGF-β; #3711) antibodies were purchased from Cell Signaling Technology (Danvers, MA, United States). Mouse anti-arginase-1 (Arg-1; #sc-271430), mouse anti-IL-10 (#sc-8438), rabbit anti-nuclear factor erythroid 2-related factor 2 (Nrf2; #sc-365949), mouse anti-β-actin (#sc-47778), rabbit anti-nuclear factor kappa-light-chain-enhancer of activated B cells (NF-κB; #sc-7151), and rabbit anti-p-NF-κB (#sc-101752) antibodies were purchased from Santa Cruz Biotechnology (Santa Cruz, CA, United States). Mouse anti-cyclooxygenase-2 (COX-2; #610203), mouse anti-inducible nitric oxide synthase (iNOS; #610329), mouse anti-dopamine- and cAMP-regulated neuronal phosphoprotein-32 (DARPP-32; #611520), and rat anti-tumor necrosis factor-alpha (TNF-α; #551225) antibodies were purchased from BD Biosciences (San Jose, CA, United States). Mouse anti-heme oxygenase-1 (HO-1; #ADI-OSA-110) antibody was purchased from Invitrogen (Waltham, MA, United States). Rabbit anti-ionized calcium-binding adapter molecule 1 (Iba-1; #NCNP24) was purchased from Wako Pure Chemical (Osaka, Japan). Goat anti-4-hydroxynonenal (4-HNE; #HNE11-S) was purchased from Alpha Diagnostic International (San Antonio, TX, United States). Mouse anti-heme oxygenase-1 (HO-1; #ADI-OSA-110) was purchased from Enzo Biochem (Farmingdale, NY, United States). Rat anti-cluster of differentiation 68 (CD68; #FA-11) antibody was purchased from ALZFORUM (Madrid, Spain). MitoSOX™ Red mitochondrial superoxide indicator (#M36008) was purchased from Molecular Probes (Eugene, OR, United States). All other reagents were purchased from Sigma-Aldrich (St. Louis, MO, United States).

### 2.2 Preparation of SOSC

**FIGURE 1:**
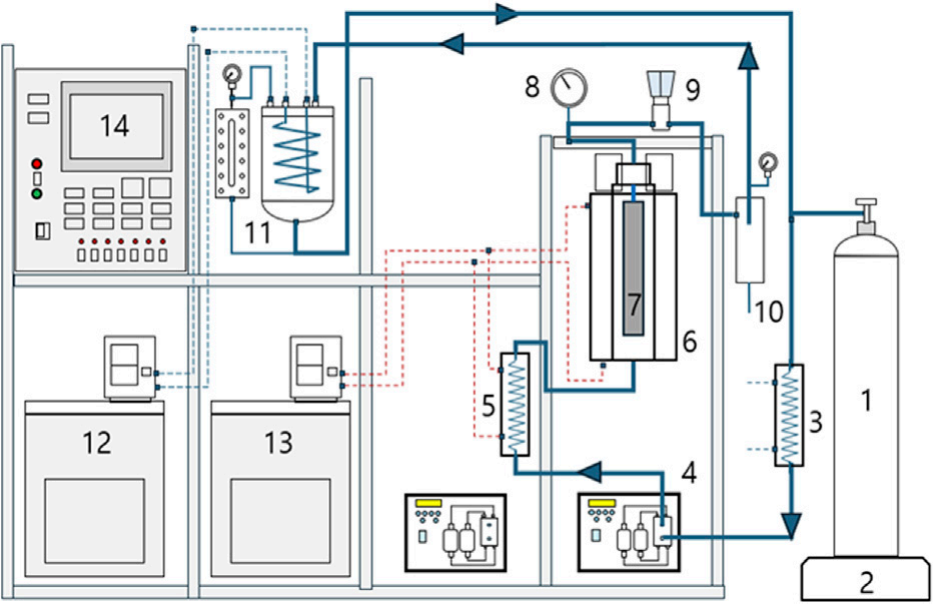
A schematic diagram of the supercritical extraction system: 1, CO_2_ cylinder; 2, Balance; 3, Precooler; 4, Plunger pump; 5, Preheater; 6, Extractor vessel; 7, Inner basket; 8, Pressure gauge; 9, Back Pressure Regulator; 10, Separator; 11, CO_2_ storage with level gauge; 12, Cooling Bath Circulator; 13, Heating Bath Circulator; 14, Process Control System.

SSC was provided by Mungyeong-si Distribution Corporation (Mungyeong, Republic of Korea). SSC samples were ground to an average particle size of 520 μm. Commercial-grade carbon dioxide (purity 99.5%) was purchased from a local gas company (Seoul, Republic of Korea). Supercritical CO2 extraction experiments were carried out using a custom-built high-pressure extraction apparatus. Figure 1 shows a schematic of the extraction system. The apparatus consisted mainly of a high-pressure extraction vessel (6), a back-pressure regulator (9), a separator (10), a liquid CO2 storage tank (11), a high-pressure CO2 pump (4), a precooler (Landis, Amara et al.), and a preheater (5). The extraction vessel was equipped with an internal basket of 250 mL (33.5 mm inner diameter × 284 mm height). The internal basket had a bottom with porous, sintered steel plates to support feed materials. The extraction vessel was rated for a maximum pressure of 400 bar at 80°C. The separator volume was 127 mL (30 mm ID × 180 mm H). The volume of the CO2 recycle storage tank was 4,600 mL (150 mm ID × 260 mm H).

A back-pressure regulator was purchased from HIFLUX Co., Ltd. (Daejeon, Republic of Korea). It was rated for a maximum pressure of 41.3 MPa. Supercritical extraction was carried out at 10–40 MPa in a temperature range of 40°C–60°C. The temperature in the extraction vessel was controlled with an external heating jacket through which water at the desired temperature was circulated. After 50 g of ground SSC was loaded into the internal basket, the basket was aligned with the extraction vessel cover. A plunger pump (HKS-3000, Hanyang Accuracy, Republic of Korea) was used to initially fill the vessel to the desired extraction pressure and then circulate CO2 in an up-flow direction through the system. Extracts were collected at a constant flow rate of 30 mL CO2/min for 1 hour. At the end of the extraction, the weight ratio of CO2 to SSC was approximately 25. The extractor was depressurized to unload the residue.

### 2.3 High-performance liquid chromatography (HPLC) analysis of SOSC

The SOSC was standardized with schizandrin, gomisin A, and gomisin N for quality assurance. SOSC (6.0 mg) was dissolved with tetrahydrofuran (1.0 mL). Standard compounds were isolated as described in our previous study (Jeong et al., 2017). Briefly, the purity of each standard compound was confirmed to be over 98% by HPLC and nuclear magnetic resonance (NMR) analysis. Standard compounds were prepared by serial dilution of methanol (62.5, 125, 250, 500, and 1,000 μg/mL). Each solution was filtered through a 0.20 μm syringe filter (Whatman Inc., Maidstone, United Kingdom) before HPLC analysis. The analysis was performed using Waters Alliance 2,795 and Waters 996 PDA. The analysis was carried out with an analytical YMC-Triart C18 column (YMC, 150 × 4.6 mm I.D., 5 μm). Mobile phase solvents A (water contained 0.1% formic acid) and B (methanol contained 0.1% formic acid) were operated with a gradient elution at a flow rate of 0.5 mL/min as follows: 76%–77% B (0–40 min), 77%–100% B (45–50 min) and then held for 10 min before returning to initial conditions. The injection volume was 10 μL. UV detection was conducted at 254 nm. The column temperature was 25°C. The analysis was repeated three times.

### 2.4 Animals and ethical approval

Male adult C57BL/6 mice (Narabiotec Co., Ltd., Seoul, Republic of Korea; weight, 23–25 g; n = 105; Seed mice were originated from Taconic Biosciences Inc., Rensselaer, NY, United States) were kept under constant temperature (23 ± 2°C) and humidity (55 ± 5%) conditions with a 12-h light-dark cycle (light on 08:00 to 20:00), and fed food and water *ad libitum*. The mice were allowed to habituate to the housing facilities for 1 week before the experiments. All experimental procedures were reviewed and approved by the Institutional Animal Care and Use Committee of Kyung Hee University (KHSASP-22–032). Proper randomization of mice and handling of data were performed in a blinded manner in accordance with recommendations from a NIH Workshop on preclinical models of neurological diseases (Landis et al., 2012).

### 2.5 Experimental groups, model induction, and SOSC administration

The experimental group was divided into the following groups: The Sham group [saline treatment, i. p. + vehicle, p. o.], 3-NPA [3-NPA, i. p. + vehicle, p. o.], 3-NPA + SOSC75 group [3-NPA, i. p. + 75 mg/kg of SOSC, p. o.], 3-NPA + SOSC150 group [3-NPA, i. p. + 150 mg/kg of SOSC, p. o.], 3-NPA + SOSC300 group [3-NPA, i. p. + 300 mg/kg of SOSC, p. o.], and SOSC alone group [saline treatment, i. p. + 300 mg/kg of SOSC, p. o.]. 3-NPA model was induced according to the previous described (Jang and Cho, 2016; Jang et al., 2014; Jang et al., 2013; Kim et al., 2017). Briefly, 3-NPA solution was given intraperitoneally (i.p.) twice daily for 2 days at 12-h intervals (8:00 a.m. and 8:00 p.m.) at a dose of 60 mg/kg on the first day and 80 mg/kg on the second day (60–60–80–80 dose regimen). SOSC was prepared with a vehicle [1% tween 80 in phosphate-buffered saline (PBS)] and administered at different doses (75, 150, and 300 mg/kg) to determine the most effective dose. Total daily dose of SOSC for mice was determined by formula for dose translation based on body surface area (Reagan-Shaw et al., 2008) after considering body weight of animals, final extract yield, and traditional dose in humans. SOSC was administered at 1 hour before 3-NPA intoxication. Experiment was repeated at least three times using the same protocol.

### 2.6 Semi-quantitative behavioral assessment and standard motor behavioral tests

The severity of neurological impairment (motor disability) induced by 3-NPA was assessed by an experimenter who was unaware of the experimental condition under constant temperature (23 ± 2°C) and humidity (55 ± 5%) in a quiet room using the behavioral scale as previously described (Choi et al., 2018; Fernagut et al., 2002; Jang and Cho, 2016; Jang et al., 2019; Jang et al., 2014; Jang et al., 2013; Jang et al., 2018; Kim et al., 2017). Briefly, mice (n = 14 per group, consisting of 2 sets of 7 mice per group) were assessed for behavioral semi-quantitative evaluation 24 h after the last (4^th^) 3-NPA intoxication. The three-level (0, 1, and 2) scale was used to measure the severity of the following five items (maximal score = 10), which constituted the main motor symptoms observed: hindlimb clasping, global activity in a free-moving environment, hindlimb dystonia, truncal dystonia (kyphotic posture), and balance adjustment to a postural challenge. Standardized motor behavioral tests (pole and rotarod tests) were conducted as previously described (Choi et al., 2018; Jang et al., 2018). Briefly, mice (n = 7 per group) were assessed using the pole and rotarod tests 24 h after the first 3-NPA intoxication. For the pole test, each mouse was placed at the top of a pole with a rough surface (1 cm in diameter and 50 cm in height), positioned with its head facing upward. The time taken for the mouse to completely turn downward at the top of the pole and climb down to the floor was recorded. One hour after the pole test, each mouse was placed on a rotating rod (diameter = 4 cm) set to rotate at 16 rpm, and performance was tested for 5 min. The latency to fall off the rotarod apparatus during this time was recorded using magnetic trip plates. Mice were acclimated to both the pole and rotarod apparatuses for 5 days prior to the first test. Mice that successfully turned downward at the top of the pole and climbed down to the floor within 2 min, as well as those that remained on the rod without falling during a 5-min training session, were selected and randomly assigned to experimental groups.

### 2.7 Histopathological analysis of striatal damage

To investigate the histopathological alterations of the striatum following 3-NPA intoxication, we used a previously described protocol (Jang et al., 2019; Kim et al., 2017). Briefly, 24 h after the last (4^th^) 3-NPA intoxication, the mice (n = 5 per group) were anesthetized with isoflurane and then perfused intracardially with saline and iced 4% paraformaldehyde in 0.1 M phosphate buffer (PB, pH 7.4). Sequential coronal sections (30-μm thickness) were acquired from the corpus callosum throughout the entire striatum (bregma 1.40 ∼ −1.30 mm) using published method (Franklin and Paxinos, 2008). Free-floating sections were collected in an antifreeze solution (30% sucrose in PBS) and stored at −20°C. The stained sections from the level of the mid-striatum were captured using a digital camera (Olympus DP-70) and the mean level of lesion area to whole striatal area was analyzed using the NIH ImageJ program (http://rsbweb.nih.gov/ij/).

### 2.8 Immunofluorescence evaluation

Immunofluorescence analysis was performed as previously described (Jang et al., 2019; Lee et al., 2016; Lee et al., 2018). Briefly, 24 h after the last (4^th^) 3-NPA intoxication, free floating brain sections (30-μm thickness; 3 sections per brain) from all groups (n = 5 per group) were incubated with rabbit anti-cleaved caspase-3 (1:500; Cell Signaling Technology), rabbit anti-ionized calcium-binding adapter molecule (Iba)-1 (1:2,000; WAKO, Chuo-Ku, Japan), mouse anti-Nrf2 (1:1,000; Santa Cruz Biotechnology, or mouse anti-HO-1 (1:1,000), or goat anti-4-HNE (1:1,000; Alpha Diagnostic International Inc.), mouse anti-Arg-1 (1:1,000; Santa Cruz Biotechnology), mouse anti-DARPP32 (1:1,000; BD Biosciences), mouse anti-HO-1 (1:1,000; Enzo), mouse anti-interleukin-10 (IL-10; 1:1,000; Santa Cruz Biotechnology), mouse anti-Nrf2 (1:1,000; Santa Cruz Biotechnology), rabbit anti-cleaved caspase-3 (1:500; Cell Signaling Technology), rabbit anti-ERK (1:1,000; Cell Signaling Technology), rabbit anti-Iba-1 (1:500; WAKO), rabbit anti-IκB (1:1,000; Cell Signaling Technology), rabbit anti-interleukin-1 beta (IL-1β; 1:1,000; Cell Signaling Technology), rabbit anti-JNK (1:1,000; Cell Signaling Technology), rabbit anti-NF-κB p65 (1:1,000; Santa Cruz Biotechnology), rabbit anti-p38 (1:1,000; Cell Signaling Technology), rabbit anti-p-ERK (1:1,000; Cell Signaling Technology), rabbit anti-p-IκB (1:1,000; Cell Signaling Technology), rabbit anti-p-JNK (1:1,000; Cell Signaling Technology), rabbit anti-p-NF-κB p65 (1:500 Santa Cruz Biotechnology), rabbit anti-p-p38 (1:1,000; Cell Signaling Technology), rabbit anti-pro-caspase-3 (1:1,000; Cell Signaling Technology), or rat anti-CD68 (1:1,000; ALZFORUM) antibody. And region of interest of each section were captured using a confocal laser scanning microscope (Carl Zeiss LSM 5 PASCAL). And then the intensity of Iba-1 (+) cells and the number of Nrf2 (+) cells and HO-1 (+) cells per striatum were manually and blindly measured.

### 2.9 Western blot analysis

Western blot analysis was performed using previously published method (Jang et al., 2019; Kim et al., 2017). Briefly, 24 h after the last (4^th^) 3-NPA intoxication, the striatal proteins from all groups (n = 5 per group) were incubated with primary antibodies including goat anti-4-HNE (1:1,000; Alpha Diagnostic International Inc.), mouse anti-Arg-1 (1:500; Santa Cruz Biotechnology), mouse anti-DARPP32 (1:1,000; BD Biosciences), mouse anti-HO-1 (1:1,000; Enzo), mouse anti-IL-10 (1:500; Santa Cruz Biotechnology), mouse anti-Nrf2 (1:1,000; Santa Cruz Biotechnology), rabbit anti-cleaved caspase-3 (1:500; Cell Signaling Technology), rabbit anti-ERK (1:1,000; Cell Signaling Technology), rabbit anti-Iba-1 (1:500; WAKO), rabbit anti-IκB (1:1,000; Cell Signaling Technology), rabbit anti-IL-1β (1:1,000; Cell Signaling Technology), rabbit anti-JNK (1:1,000; Cell Signaling Technology), rabbit anti-NF-κB p65 (1:1,000; Santa Cruz Biotechnology), rabbit anti-p38 (1:1,000; Cell Signaling Technology), rabbit anti-p-ERK (1:1,000; Cell Signaling Technology), rabbit anti-p-IκB (1:1,000; Cell Signaling Technology), rabbit anti-p-JNK (1:1,000; Cell Signaling Technology), rabbit anti-p-NF-κB p65 (1:500 Santa Cruz Biotechnology), rabbit anti-p-p38 (1:1,000; Cell Signaling Technology), rabbit anti-pro-caspase-3 (1:1,000; Cell Signaling Technology), rabbit anti-transforming growth factor-beta (TGF-β; 1:1,000; Cell Signaling Technology), and rat anti-CD68 (1:1,000; ALZFORUM) antibodies. For normalization of antibody signals, membranes were stripped and reprobed with antibodies against glyceraldehyde-3-phosphate dehydrogenase (GAPDH; 1:5,000; Cell Signaling Technology), beta-Actin (β-Actin; 1:5,000; Santa Cruz Biotechnology) and total IκB/NF-κB p65/JNK/ERK/p38 (1:1,000; Cell Signaling Technology). Data are expressed as the ratio of corresponding protein signal to GAPDH or total protein signal for each sample.

### 2.10 Reverse transcription polymerase chain reaction (RT-PCR) analysis

To measure the mRNA level of inflammatory factors, 24 h after the last (4^th^) 3-NPA intoxication, RT-PCR analysis using the striatal lysates from all groups (n = 5 per group) was performed using PCR Master Mix as previously described (Choi et al., 2018). Expression levels of each gene were normalized to those of GAPDH. All PCR experiments were performed at least three times, and the mean ± S.E.M. values are presented unless otherwise noted. The primer sequences were as follows: interleukin (IL)-1β-5′-TTG TGG CTG TGG AGA AGC TGT-3′ and 5′-AAC GTC ACA CAC CAG CAG GTT-3′; IL-6-5′-TCC ATC CAG TTG CCT TCT TGG-3′ and 5′-CCA CGA TTT CCC AGA GAA CAT G-3′; COX-2-5′-GCA TTC TTT GCC CAG CAC TTC ACT-3′ and 5′-TTT AAG TCC ACT CCA TGG CCC AGT-3′; iNOS-5′-GGC AAA CCC AAG GTC TAG GTT-3′ and 5′-TCG CTC AAG TTC AGC TTG GT-3′; Arg-1-5′-TCA TGG AAG TGA ACC CAA CTC TTG-3′ and 5′-TCA GTC CCT GGC TTA TGG TTA CC; IL-10–5′-GTG AAG ACT TTC TTT CAA A-3′ and 5′-TGA TCA AGA TGT CAA ACT C-3′; TGF-β-5′-CTT CAG CTC CAC AGA GAA GAA CTG C-3′ and 5′-CAC GAT CAT GTT GGA CAA CTG CTC C-3′; and GAPDH-5′-AGG TCA TCC CAG AGC TGA ACG-3′ and 5′-CAC CCT GTT GCT GTA GCC GTA T-3′.

### 2.11 3-NPA-induced BV2 cells, their conditioned medium (CM), and the activity of SH-SY5Y cells

BV2 cell lines, a type of microglial cell derived from C57BL/6 mice, were cultured in high-glucose Dulbecco’s modified Eagle’s medium (DMEM) supplemented with 10% fetal bovine serum (FBS) (Gibco, Waltham, MA). BV2 cells were maintained in a humidified incubator with 95% air and 5% CO2 at 37 °C. The medium was replaced every other day. The cytotoxic effects of schizandrin on BV2 cells were determined using two methods: direct counting of living and dead cells using a hemocytometer with an inverted microscope and the MTT (3-(4,5-dimethyl-thiazol-2-yl)-2,5-diphenyltetrazolium bromide) assay. When the cells reached a density of approximately 90%, schizandrin (40, 60, and 80 μM) was added to the cell medium. After 1 h of incubation, 3-NPA (100 μM) was added. The medium was replaced after 6 h. Cells and their cultured medium [referred to as conditioned medium (CM)] were harvested after 36 h. These cells were subjected to Western blot analysis to investigate the protein expression levels of Iba-1, p-NF-κB, and Nrf2. CM-3-NPA (CM from 3-NPA-stimulated BV2 cells) and CM-3-NPA-schizandrin (CM from 3-NPA-stimulated BV2 cells pretreated with schizandrin) were collected and used to assess the activity of SH-SY5Y cells. CM-3-NPA and CM-3-NPA-schizandrin were used to treat SH-SY5Y cells for 48 h. CM-treated SH-SY5Y cells were collected to analyze the degree of neurodegeneration (NeuN and cleaved caspase-3) via Western blot analysis. *In vitro* assays were repeated at least three times, with each experiment performed in triplicate.

### 2.12 Statistical analysis

Statistical analysis was performed using IBM SPSS Statistics version 26.0 (SPSS Inc., Chicago, United States) for Windows. Data from experiments, including behavioral tests, immunohistochemistry, Western blotting, and PCR analysis, were analyzed using the Kruskal–Wallis test (a nonparametric test) for comparisons of three or more unmatched groups. Data are presented as mean ± SEM. P-values less than 0.05 were considered statistically significant.

## 3 Results

### 3.1 HPLC analysis of SOSC

**FIGURE 2:**
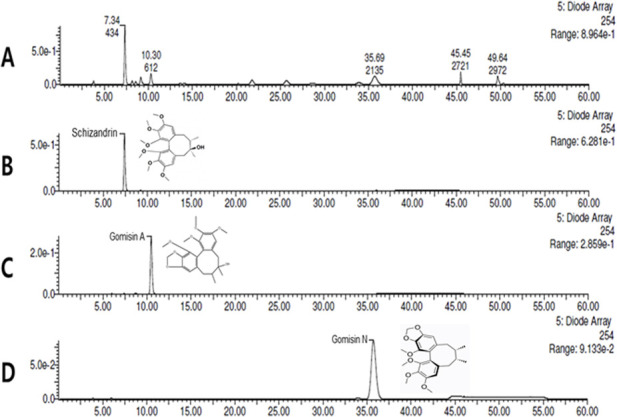
Quantitative HPLC analysis of the contents of SOSC. SOSC was standardized using schizandrin, gomisin A, and gomisin N for quality assurance. The HPLC chromatogram of SOSC revealed retention time (tR) of 7.34 min for schizandrin **(B)**, 10.30 min for gomisin A **(C)**, and 35.69 min for gomisin N **(D)** compared to reference standard peaks **(A)**. The concentrations of schizandrin, gomisin A, and gomisin N in SOSC were determined to be 54.71 ± 0.01 mg/g, 25.13 ± 0.01 mg/g, and 36.46 ± 0.01 mg/g, respectively. HPLC, high-performance liquid chromatography; SOSC, seed oil of *Schisandra chinensis.*

To determine concentrations of schizandrin, gomisin A, and gomisin N in SOSC, HPLC analysis was performed. The HPLC chromatogram of SOSC revealed a retention time (*t*_R_) of 7.34 min for schizandrin, 10.30 min for gomisin A, and 35.69 min for gomisin N when compared to a reference standard peak (Figure 2). To quantify concentrations of the three compounds, calibration curves for each compound were established using serially diluted standard solutions ranging from 1,000 to 62.5 μg/mL. Linear regression equations for these compounds exhibited high coefficients (R^2^ > 0.9990) of determination: Y = 447.17X - 5,340.1 (R^2^ = 0.9995) for schizandrin, Y = 245.81X - 592.96 (R^2^ = 0.9998) for gomisin A, and Y = 383.69X - 4,084.7 (R^2^ = 0.9994) for gomisin N. Finally, concentrations of schizandrin, gomisin A, and gomisin N in SOSC were determined to be 54.71 ± 0.01 mg/g, 25.13 ± 0.01 mg/g, and 36.46 ± 0.01 mg/g, respectively (Figure 2).

### 3.2 Ameliorative effects of SOSC on motor coordination and survival rate following 3-NPA intoxication

**FIGURE 3:**
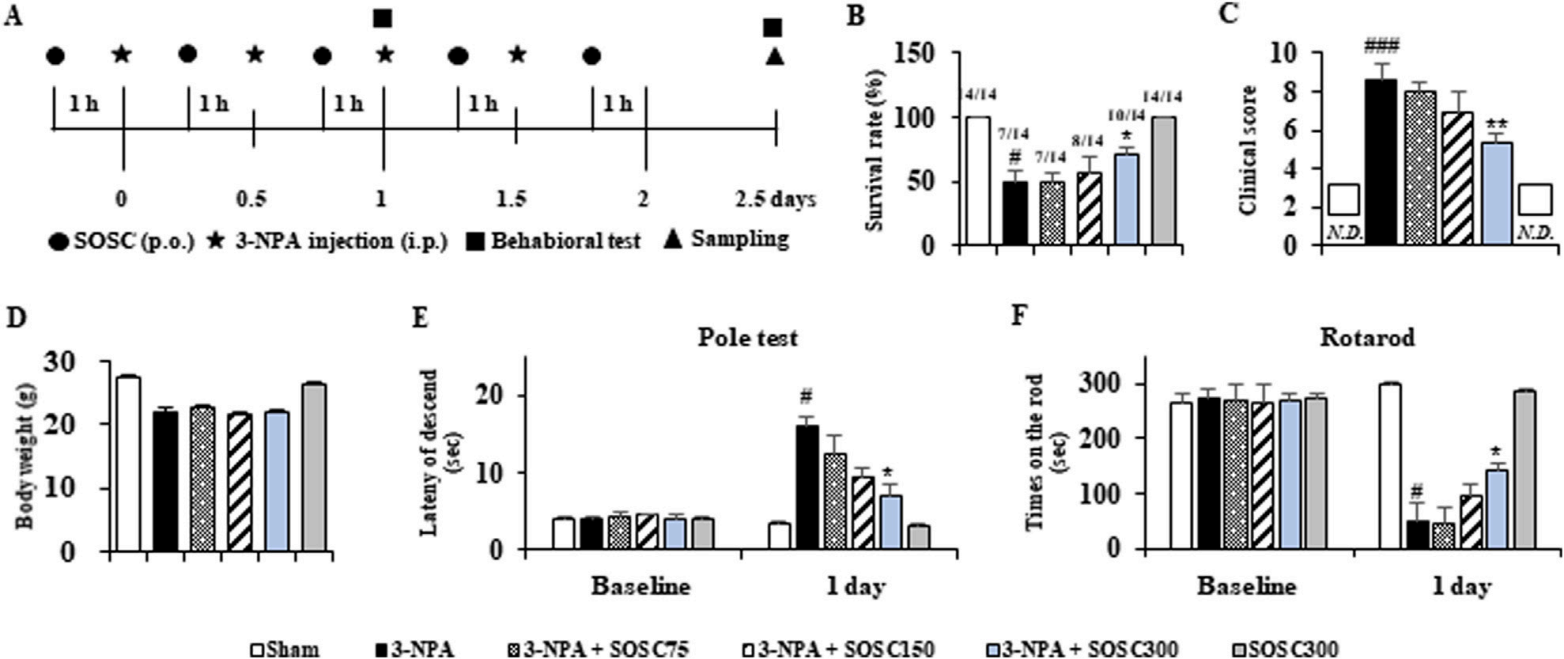
SOSC ameliorates movement dysfunction and improves survival rate after 3-NPA intoxication. **(A)** Schematic diagram of the experimental design and schedule. 3-NPA solution was administered intraperitoneally (i.p.) twice daily for 2 days at 12-h intervals at a dose of 60 mg/kg on the first day and 80 mg/kg on the second day (i.e., a 60–60–80–80 dose regimen). SOSC (75, 150, and 300 mg/kg) was administered by oral gavage once daily from 1 h before the first 3-NPA intoxication. The sham group was administered with vehicle (saline). The semi-quantitative behavioral assessment was conducted 24 h after the last (4^th^) 3-NPA intoxication, and brain (or striatum) samples were collected. Standard motor behavioral tests (pole and rotarod performance tests) were conducted 24 h after the first 3-NPA intoxication. **(B–D)** The survival rate **(B)**, movement dysfunction (clinical score for semi-quantitative behavioral assessment) **(C)**, and body weight **(D)** of mice from all groups (n = 14 per group) were measured. In Graph B, the numbers above the bars represent the number of surviving mice after the behavioral experiment divided by the number of mice at the start of the experiment. In Graph C, the squares indicate “not detected.” **(E, F)** Pole and rotarod performance tests were conducted on mice from all groups (n = 7 per group). Data are expressed as mean ± SEM (one-way ANOVA with *post hoc*; #*p* < 0.05, ##*p* < 0.01, and ###*p* < 0.001 vs. Sham group; *p < 0.05, **p < 0.01, and ***p < 0.001 vs. 3-NPA group). 3-NPA, 3-nitropropionic acid; SEM, standard error of the mean; SOSC, seed oil of *Schisandra chinensis.*

First, we determined whether SOSC could affect movement disorder and survival rate of mice after 3-NPA-intoxication (Figure 3A). The survival rate at the end of two representative experimental sets increased to 71.4% (n = 10/14) in the 3-NPA + SOSC 300 mg/kg/day group, compared to 50.0% (n = 7/14) in the 3-NPA group (Figure 3B). Twenty-four hours after the last (4^th^) intoxication of 3-NPA, mice displayed severe neurological dysfunction (score, 8.7 ± 0.8). However, mice in the 3-NPA + SOSC group displayed significantly lower neurological scores (5.4 ± 0.4 for SOSC 300 mg/kg/day groups) than those in the 3-NPA group (Figure 3C). The mean loss of BW was significantly alleviated by 3-NPA. However, it was not significantly restored by SOSC administration (Figure 3D). SOSC alone (300 mg/kg/day) administration did not significantly affect neurological score, survival rate, or BW of normal mice (Figures 3A–D). To emphasize the efficacy of SOSC on motor coordination, the pole test and rotarod test were additionally conducted. All mice in each group (n = 7 per group) survived up to the first day following 3-NPA intoxication, during which the two behavioral tests were performed (Figures 3E, F). In the pole test, the average descent time to the bottom of the pole increased in the 3-NPA group (16.1 ± 1.2 s) compared to the sham group (3.2 ± 0.2 s). However, the average descent time was reduced to 7.3 ± 1.8 s in the SOSC 300 mg/kg/day group, compared to 16.1 ± 1.2 s in the 3-NPA group (Figure 3E). In the rotarod test, the average latency to fall decreased in the 3-NPA group (50.4 ± 3.6 s) compared to the sham group (287.0 ± 4.9 s). However, the average latency improved to 141.2 ± 14.8 s in the SOSC 300 mg/kg/day group compared to that in the 3-NPA group (Figure 3F). SOSC alone (300 mg/kg/day) administration did not significantly affect the pole test and rotarod test of normal mice (Figures 3E, F).

### 3.3 Ameliorative effects of SOSC on striatal neurodegeneration and apoptosis following 3-NPA intoxication

**FIGURE 4:**
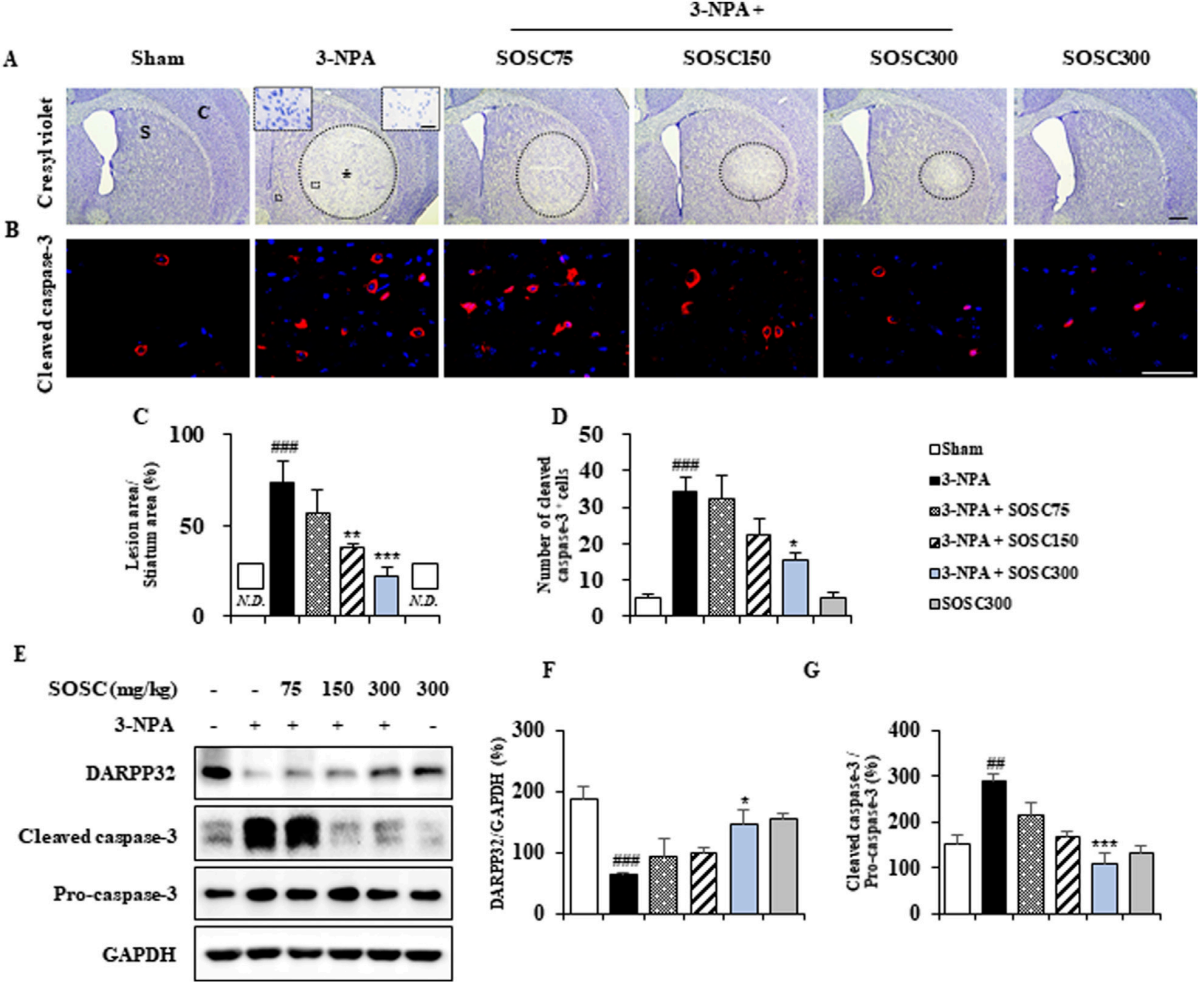
SOSC inhibits neurodegeneration and apoptosis in the striatum after 3-NPA intoxication. **(A–D)** Twenty-four hours following the last (4^th^) 3-NPA intoxication, cryo-striatal sections (n = 3 per brain; 5 brains per group) from the sham, 3-NPA, 3-NPA + SOSC75 (75 mg/kg/day), 3-NPA + SOSC150 (150 mg/kg/day), 3-NPA + SOSC300 (300 mg/kg/day), and SOSC (300 mg/kg/day) groups were subjected to histopathological staining using cresyl violet dye **(A)** and immunofluorescence staining with a cleaved caspase-3 antibody **(B)** to investigate levels of striatal cell death and apoptosis. The ratio of the mean lesion area to the entire striatum **(C)** and the number of cleaved caspase-3-positive cells per striatum **(D)** were quantified. In panel A, the left and right insets of the 3-NPA group are magnifications of the squares in the original image, showing the live cell region and the dead cell region, respectively. **(E–G)** At 24 h following the last (4^th^) 3-NPA intoxication, striatal lysates from all groups were subjected to Western blot analysis using DARPP32 and cleaved caspase-3 antibodies **(E)**, and the results were quantified **(F, G)**, respectively. Scale bar = 100 μm in A, 20 μm in B, and 20 μm in insets. N.D., not detected. Data are expressed as mean ± SEM (one-way ANOVA with *post hoc*; #*p* < 0.05, ##*p* < 0.01, and ###*p* < 0.001 vs. Sham group; *p < 0.05, **p < 0.01, and ***p < 0.001 vs. 3-NPA group). 3-NPA, 3-nitropropionic acid; Asterisk, striatal lesions; C, normal cortex; GAPDH, glyceraldehyde-3-phosphate dehydrogenase; S, normal striatum; SEM, standard error of the mean; SOSC, seed oil of *Schisandra chinensis.*

3-NPA produces striatal neurodegeneration and some neurological disturbances known to be characteristics of Huntington’s disease in rodents and primates (Brouillet et al., 2005; Tunez et al., 2010; Upadhayay et al., 2023). Thus, we explored whether SOSC could ameliorate striatal neurodegeneration by 3-NPA-intoxication. Twenty-four hours after the last (4^th^) 3-NPA-intoxication, coronal cryo-sections of brain (n = 5 group) including the striatum were stained with cresyl violet dye. Figure 4A shows representative striatal images from sham, 3-NPA, 3-NPA + SOSC (75, 150, and 300 mg/kg/day), and SOSC alone (300 mg/kg/day) groups. The 3-NPA-treated group exhibited visible bilateral striatal lesions (pale areas surrounded by dotted line), whereas treatment with SOSC resulted in smaller lesions in a dose-dependent manner (Figure 4A). Results of quantification revealed that the ratio of mean lesion area to the entire striatum was 73.2% in the 3-NPA group, while this ratio was reduced by SOSC in dose-dependent manner to 56.4%, 37.6%, and 21.8% in the group administered with SOSC at 75, 15, and 300 mg/kg/day, respectively (Figure 4C). Based on results from cresyl violet staining (Figures 4A, C), to further compare levels of neurodegeneration, striatal lysates of all groups were subjected to Western blot assay with DARPP32 antiserum (a marker for medium spiny neurons of the striatum) (Ivkovic et al., 1997) (Figures 4B, E, F). Protein expression of DARPP32 was clearly decreased to 62.6 ± 3.3 in the 3-NPA group compared with the Sham group (188.6 ± 19.4), whereas its expression was significantly restored to 147.5 ± 23.4 in the 300 mg/kg/day SOSC-administered 3-NPA group (Figures 4E, F). To test whether the anti-neurodegenerative effect of SOSC might be related to apoptosis, we investigated the immunoreactive level of cleaved caspase-3 (a representative apoptosis marker) in the striatum by immunofluorescence staining (Figures 4B–D). Cleaved caspase-3 immunoreactivity was increased in the 3-NPA group than that in the sham group. However, its immunoreactivity was decreased by SOSC (at 75, 15, and 300 mg/kg/day) in a dose-dependent manner (Figures 4B, D). The expression pattern of cleaved caspase-3 was similar to that of cleaved caspase-3 based on Western blot (Figures 4E, G). Since administration with 300 mg/kg/day of SOSC was the most effects on behavioral dysfunction (Figure 3B), survival rate (Figure 3C), and striatal neurodegeneration (Figures 4A–G) following 3-NPA-intoxication, this dose of SOSC was used in further studies.

### 3.4 Ameliorative effects of SOSC on microglial activation in the striatum following 3-NPA intoxication

**FIGURE 5:**
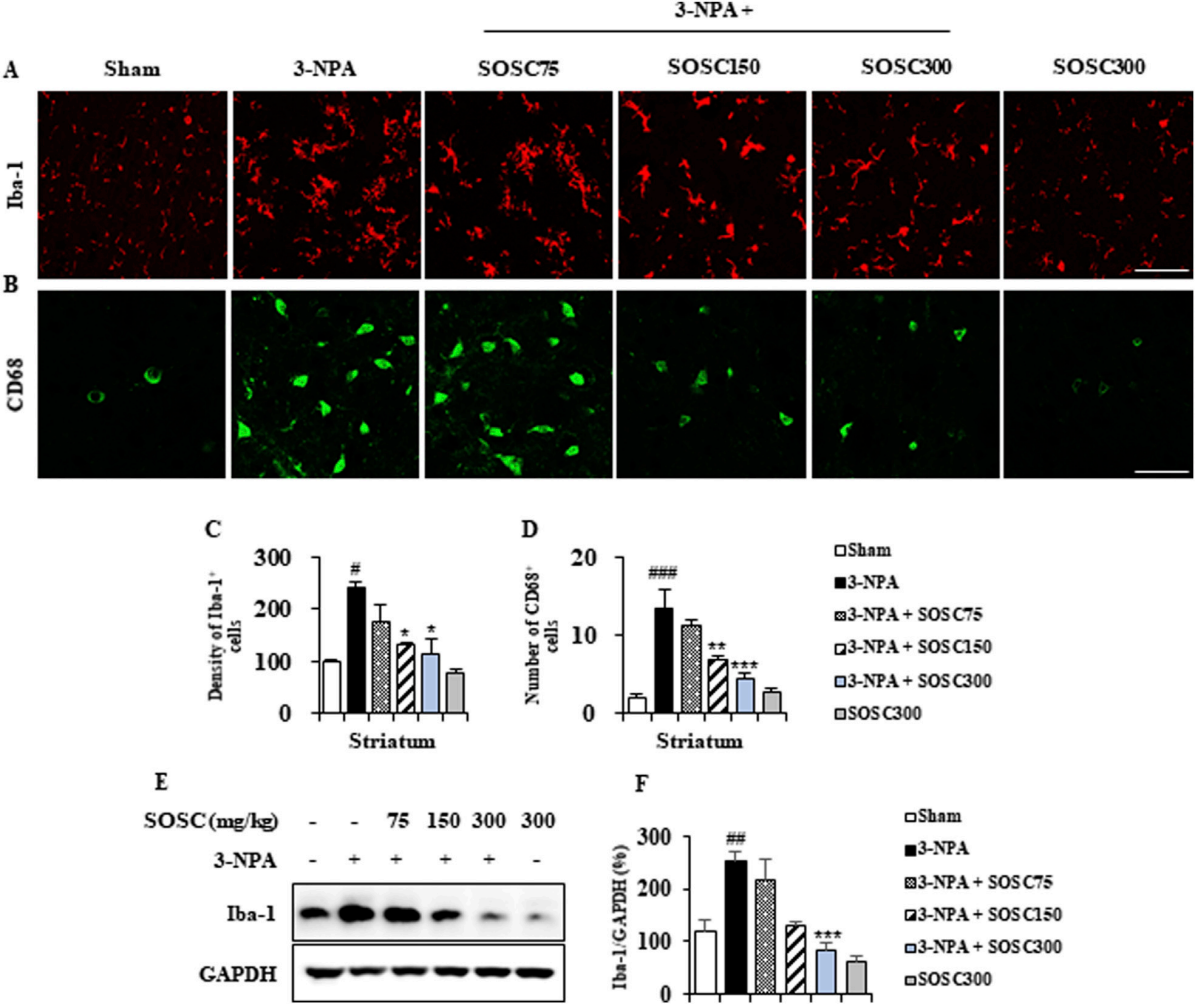
SOSC suppresses microglial migration and activation in the striatum after 3-NPA intoxication. **(A–D)** Twenty-four hours after the last (4^th^) 3-NPA intoxication, striata (n = 5 per group) from the sham, 3-NPA, 3-NPA + SOSC75 (75 mg/kg/day), 3-NPA + SOSC150 (150 mg/kg/day), 3-NPA + SOSC300 (300 mg/kg/day), and SOSC (300 mg/kg/day) groups were used to investigate levels of microglial migration and activation. Cryo-striatal sections from all groups were subjected to immunofluorescence staining using the Iba-1 antibody. **(A)** The intensity of Iba-1-positive cells in the striatum was measured. **(B)** Striatal lysates from all groups were subjected to Western blot analysis using the Iba-1 antibody **(C)** and quantified **(D)**. **(E, F)** At 24 h following the last (4^th^) 3-NPA intoxication, striatal lysates from all groups were subjected to Western blot analysis using Iba-1 antibody **(E)** and the result was quantified (F). Scale bar = 20 μm. Data are expressed as mean ± SEM (one-way ANOVA with *post hoc*; #*p* < 0.05, ##*p* < 0.01, and ###*p* < 0.001 vs. the sham group; *p < 0.05, **p < 0.01, and ***p < 0.001 vs. the 3-NPA group). 3-NPA, 3-nitropropionic acid; GAPDH, glyceraldehyde 3-phosphate dehydrogenase; SOSC, seed oil of *Schisandra chinensis*.

Microglia can migrate into/around/within lesions of brain with neurodegenerative diseases including HD (Deczkowska et al., 2018; Gao et al., 2023; Kumari and Gensel, 2023). They become activated following exposure to pathogen-associated molecular patterns (PAMPs), endogenous damage-associated molecular patterns (DAMPs), and removal of immune-suppressive signals. Activated microglia can acquire different phenotypes (pro- and anti-inflammatory) depending on cues in its surrounding environment (Deczkowska et al., 2018; Gao et al., 2023; Kumari and Gensel, 2023). Thus, we investigated whether SOSC could inhibit microglial activation in striatal lesions from all groups (n = 5 per group) after 3-NPA-intoxication (Figures 5A–D). In striatal sections of the 3-NPA group, Iba-1 (a marker for microglia/macrophage lineage cells)-immunoreactive cells revealed morphology of the activated type with bigger cell bodies and extended (short and thick) processes than those in the sham group, which displayed typical forms of resting cells including relatively small soma and long thin processes (Deczkowska et al., 2018; Gao et al., 2023; Kumari and Gensel, 2023) (Figures 5A–C). However, the mean level of Iba-1-immunoreactive cells to total striatal area was remarkably reduced in striatal sections of the 3-NPA + SOSC group than in the 3-NPA group (Figures 5A, B), in agreement with altered protein expression of Iba-1 (by 251.3% in the 3-NPA group; by 215.6%, 128.3%, and 83.5% in 75, 150, and 300 mg/kg SOSC groups, respectively) based on Western blot analysis (Figure 5C). The normal morphology of Iba-1 immunoreactive cells based on immunofluorescence and normal Iba-1 protein expression based on Western blot analysis were not significantly affected by administration with SOSC (300 mg/kg/day) alone (Figures 5A–D). These findings suggest that SOSC might inhibit microglial migration and activation. Such inhibition is closely associated with reduction of striatal cell death and mitigation of neurological impairment following 3-NPA-intoxication.

### 3.5 Ameliorative effects of SOSC on pro- and anti-inflammatory mediators in the striatum following 3-NPA intoxication

**FIGURE 6:**
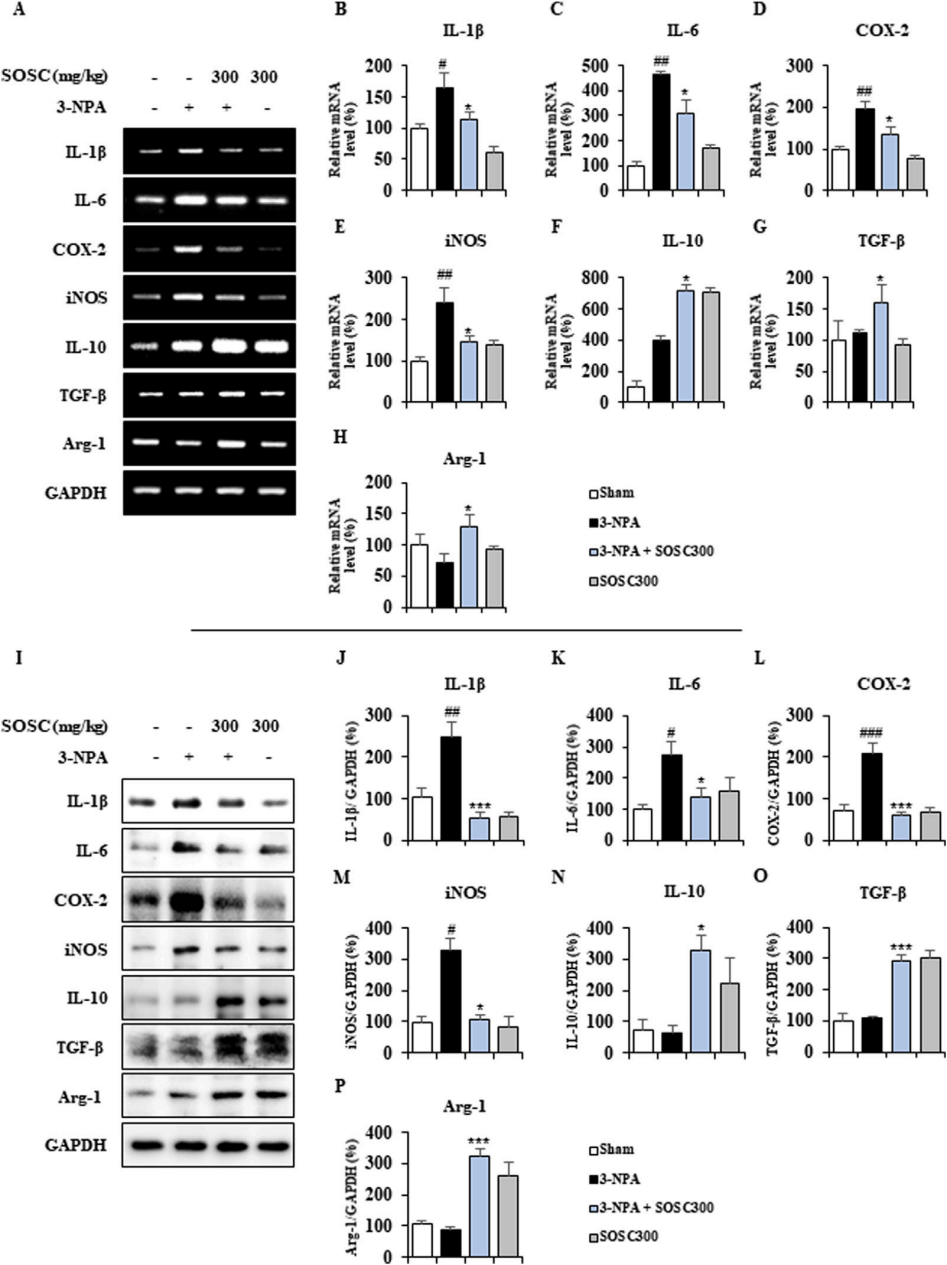
SOSC regulates mRNA levels of pro- and anti-inflammatory mediators in the striatum after 3-NPA intoxication. **(A–H)** Twelve hours after the last (4^th^) 3-NPA intoxication, striatal lysates (n = 5 per group) from sham, 3-NPA, 3-NPA + SOSC (300 mg/kg/day), and SOSC (300 mg/kg/day) groups were subjected to RT-PCR **(A)** and quantified **(B–I)**. SOSC reduced mRNA expression levels of representative pro-inflammatory cytokines [IL-1β **(A, B)** and IL-6 **(A, C)**] and enzymes [COX-2 **(A, D)** and iNOS **(A, E)**], but enhanced mRNA expression levels of representative anti-inflammatory cytokines [IL-10 **(A, F)** and TGF-β **(A, G)**] and mediators [arginase-1 **(A, H)**]. **(I–P)** Twelve hours after the last (4^th^) 3-NPA intoxication, striatal lysates (n = 5 per group) from all groups were subjected to Western blot analysis **(I)** and quantified **(J–P)**. SOSC reduced protein expression levels of representative pro-inflammatory cytokines [IL-1β **(I, J)** and IL-6 **(I, K)**] and enzymes [COX-2 **(I, L)** and iNOS **(I, M)**], but enhanced protein expression levels of representative anti-inflammatory cytokines [IL-10 **(I, N)** and TGF-β **(I, O)**] and mediators [arginase-1 **(I, P)**]. Data are expressed as mean ± SEM (one-way ANOVA with *post hoc*; #*p* < 0.05, ##*p* < 0.01, and ###*p* < 0.001 vs. Sham group; *p < 0.05, **p < 0.01, and ***p < 0.001 vs. 3-NPA group). 3-NPA, 3-nitropropionic acid; ARG1, arginase-1; COX-2, cyclooxygenase-2; GAPDH, glyceraldehyde 3-phosphate dehydrogenase; IL, interleukin; iNOS, inducible nitric oxide synthase; SOSC, seed oil of *Schisandra chinensis*; TGF-β, transforming growth factor-β.

Activated microglia around (or within) CNS lesions can secrete pro- or anti-inflammatory enzymes and cytokines that are either beneficial or detrimental to neuronal survival and cell death signaling pathways (Ferger et al., 2010; Lobsiger and Cleveland, 2007; Pavese et al., 2006). Thus, we determined whether downregulation of microglial activation by SOSC might produce alteration in mRNA expression of representative pro-inflammatory and anti-inflammatory mediators by RT PCR analysis (Figures 6A–H). mRNA expression levels of pro-inflammatory cytokines (IL-1β and IL-6) and enzymes (COX-2 and iNOS) were enhanced in the 3-NPA group than in the sham group with the following results: IL-1β increased by 165.6%, IL-6 increased by 464.2%, COX-2 increased by 197.5%, and iNOS increased by 238.6% (Figures 6A–E). However, SOSC significantly inhibited these increases induced by 3-NPA with the following results: IL-1β inhibited by 113.4%, IL-6 inhibited by 310.0%, COX-2 inhibited by 134.4%, and iNOS inhibited by 147.2% compared to those in the 3-NPA group (Figures 6A–E). On the other hand, SOSC significantly enhanced mRNA expression levels of anti-inflammatory cytokines (IL-10 and TGF-β) and anti-inflammatory agents (arginase-1) than in the 3-NPA group with the following results: IL-10 increased by 717.6%, TGF-β increased by 159.7%, and arginase-1 increased by 128.6% (Figure 6A, F–H). Additionally, to investigate whether the positive regulatory effects of SOSC on the mRNA expression levels of pro- and anti-inflammatory mediators are reflected at the protein expression level, Western blot analysis was performed for each antibody. As expected, SOSC significantly inhibited the 3-NPA-induced increase in the protein expression levels of pro-inflammatory mediators (IL-1β, IL-6, COX-2, and iNOS) (Figure 6I–M). Conversely, it significantly elevated the protein expression levels of anti-inflammatory mediators (IL-10, TGF-β, and arginase-1) compared to the 3-NPA group (Figure 6I, N–P).

### 3.6 Ameliorative effects of SOSC on inflammatory signaling pathways (MAPKs and NF-κB) in the striatum following 3-NPA intoxication

**FIGURE 7:**
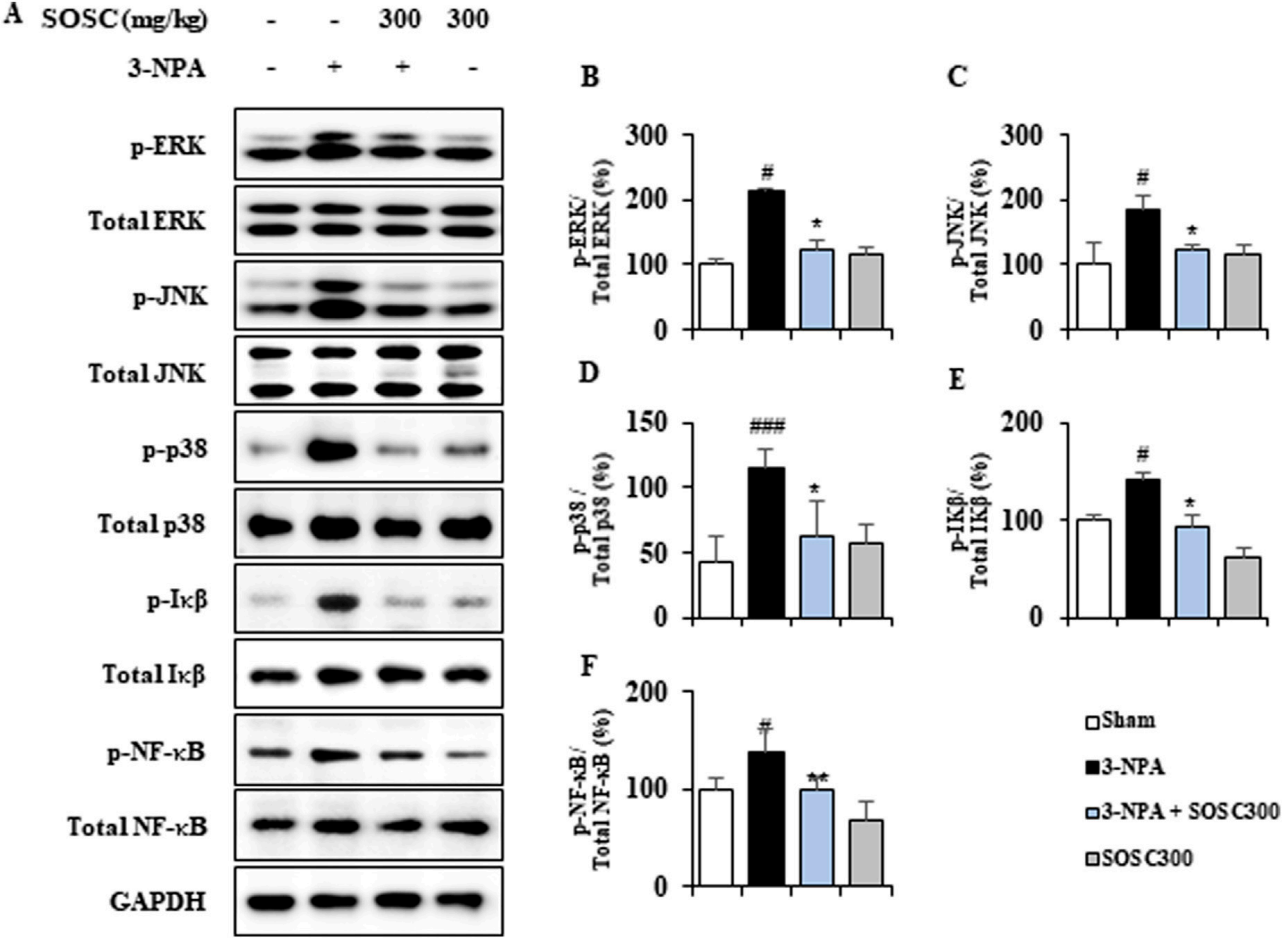
SOSC inhibits inflammatory signaling pathways (MAPKs and NF-κB) in the striatum after 3-NPA intoxication. **(A–F)** Twelve hours after the final (4^th^) 3-NPA intoxication, striatal lysates (n = 5 per group) from Sham, 3-NPA, 3-NPA + SOSC (300 mg/kg/day), and SOSC (300 mg/kg/day) groups were subjected to Western blot assay **(A)** and quantified **(B–F)**. SOSC decreased protein expression levels of p-ERK **(A, B)**, p-JNK **(A, C)**, p-p38 **(A, D)**, p-IκB (A and E), and p-NF-κB **(A, F)**. Data are expressed as mean ± SEM (one-way ANOVA with *post hoc* test; #*p* < 0.05, ##*p* < 0.01, and ###*p* < 0.001 vs. Sham group; *p < 0.05, **p < 0.01, and ***p < 0.001 vs. 3-NPA group). 3-NPA, 3-nitropropionic acid; ERK, extracellular signal-regulated kinase; GAPDH, glyceraldehyde 3-phosphate dehydrogenase; IκB, inhibitor of NF-κB; JNK, Jun amino-terminal kinases; NF-κB, nuclear factor kappa B; SOSC, seed oil of *Schisandra chinensis.*

MAPKs and NF-κB signaling pathways play critical roles in neuroinflammatory and neuropathological mechanisms of neurodegenerative disorders such as HD (Cai et al., 2022; Zhang et al., 2023). Thus, we determined whether SOSC could regulate these pathways in the striatum after 3-NPA-intoxication (Figures 7A–E). Expression levels of p-ERK, p-JNK, and p-p38 MAPKs protein were clearly increased by 213.5%, 186.2%, and 115.2%, respectively, in the striatum following the last (4^th^) 3-NPA-intoxication compared to those in the sham group. However, SOSC significantly decreased expression levels of p-ERK, p-JNK, and p-p38 MAPKs proteins by 123.7%, 124.2%, and 62.6%, respectively (Figures 7A–D). In additional, expression levels of p-IκB and p-NF-κB protein were distinguishably upregulated by 140.7% and 138.4%, respectively, in the striatum after the last (4^th^) 3-NPA-intoxication compared to those in the sham group. However, SOSC significantly downregulated their expression level in by 94.0% and 98.3%, respectively (Figures 7A, E, F). These findings suggest that SOSC could inhibit inflammatory response and striatal toxicity after 3-NPA-intoxication by inhibiting MAPKs and NF-κB signaling pathways in the striatum.

### 3.7 SOSC positively affects ROS generation and stimulates the Nrf2-HO-1 antioxidant signaling pathway in the striatum after 3-NPA intoxication

**FIGURE 8:**
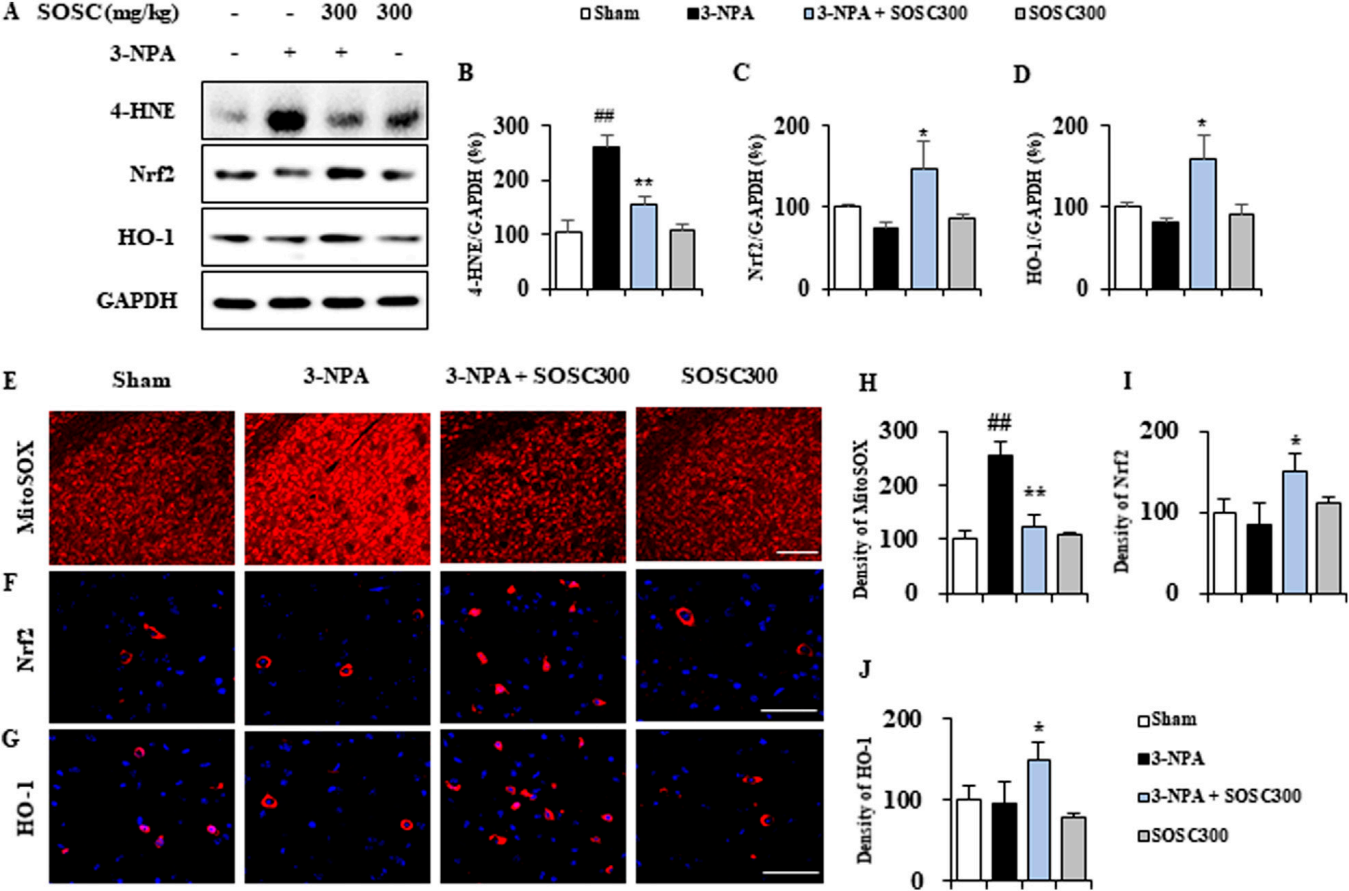
SOSC inhibits ROS and stimulates antioxidant signaling pathways (Nrf2-HO-1) in the striatum after 3-NPA intoxication. **(A–D)** Twelve hours after the last (4th) dose of 3-NPA intoxication, striatal lysates (n = 5 per group) from sham, 3-NPA, 3-NPA + SOSC (300 mg/kg/day), and SOSC (300 mg/kg/day) groups were subjected to Western blot assay **(A)** and quantified **(B–D)**. SOSC decreased the protein expression of 4-HNE (A and B) but increased the protein expression levels of Nrf2 **(A, C)** and HO-1 **(A, D)** signaling pathways. **(E–G)** Cryo-striatal sections (n = 5 per brain) from all groups (5 brains per group) were subjected to MitoSOX staining (E) and immunofluorescence staining using Nrf2 and HO-1 antibodies **(F, G)**. Scale bar = 20 μm. The intensities of the signals in the striatum were measured **(H–J)**. Data are expressed as mean ± SEM (one-way ANOVA with *post hoc*; #*p* < 0.05, ##*p* < 0.01, and ###*p* < 0.001 vs. Sham group; *p < 0.05, **p < 0.01, and *p < 0.001 vs. 3-NPA group). 3-NPA, 3-nitropropionic acid; 4-HNE, 4-hydroxynonenal; Nrf2, nuclear factor erythroid 2-related factor 2; HO-1, heme oxygenase 1; GAPDH, glyceraldehyde 3-phosphate dehydrogenase; SOSC, seed oil of *Schisandra chinensis.*

Oxidative stress has been implicated in the pathogenesis of many neurodegenerative diseases including HD (Teixeira et al., 2019). Oxidative stress leading to free radical attack on neural cells contributes calamitous role to neurodegeneration (Teixeira et al., 2019). Thus, we examined whether administration with SOSC might reduce the generation of oxidative stress in the striatum after 3-NPA-intoxication. Protein expression of 4-hydroxynonenal (4-HNE), a marker for reactive oxygen species (ROS), was clearly enhanced in the striatum of the 3-NPA group (261.5 ± 34.3) compared to that of the sham group (103.9 ± 11.6), whereas such enhanced expression was significantly reduced in the striatum of the 3-NPA + SOSC (300 mg/kg) group (156.7 ± 30.0) compared to that of the 3-NPA group (Figures 8A, B). The ameliorating effect of SOSC on 4-HNE was closely related to a downregulated intensity of MitoSOX, a mitochondrial superoxide indicator, in the striatum of 3-NPA + SOSC (300 mg/kg) group (Figure 8E). On the other side, the Nrf2/heme oxygenase 1 (HO-1) signaling pathway governs gene expression of endogenous antioxidant synthesis and ROS-eliminating enzymes as an antioxidant defense mechanism for protecting brain cells against abnormal ROS levels (Kasai et al., 2020). Thus, we further examined whether SOSC might stimulate the Nrf2/HO-1 pathway by Western blot assay for Nrf2 (a transcription factor for antioxidant responses) and HO-1 (a representative product of the Nrf2 pathway and antioxidant enzyme) (Figure 8A). Administration with SOSC (300 mg/kg) significantly stimulated Nrf2 and produced HO-1 protein by 147.5% and 159.7%, respectively, in the striatum after 3-NPA-intoxication compared to those of 3-NPA group without administration with SOSC (74.8 ± 7.5 and 82.4 ± 5.1, respectively) (Figures 8A, C, D, F). Stimulated effects of SOSC on Nrf2 and HO-1 proteins were closely connected with enhanced nuclear translocation of Nrf2 and enhanced cytoplasmic expression of HO-1 in the striatum of the 3-NPA + SOSC (300 mg/kg) group compared to those of the 3-NPA group without administration with SOSC (Figures 8G, H). These results suggest that SOSC might have improved striatal neurodegeneration by inhibiting ROS generation and stabilizing the Nrf2/HO-1 antioxidant signaling pathway in the brains of the 3-NPA group.

### 3.8 Anti-apoptotic effects of microglial conditioned medium containing schizandrin in 3-NPA-induced SH-SY5Y cells

**FIGURE 9:**
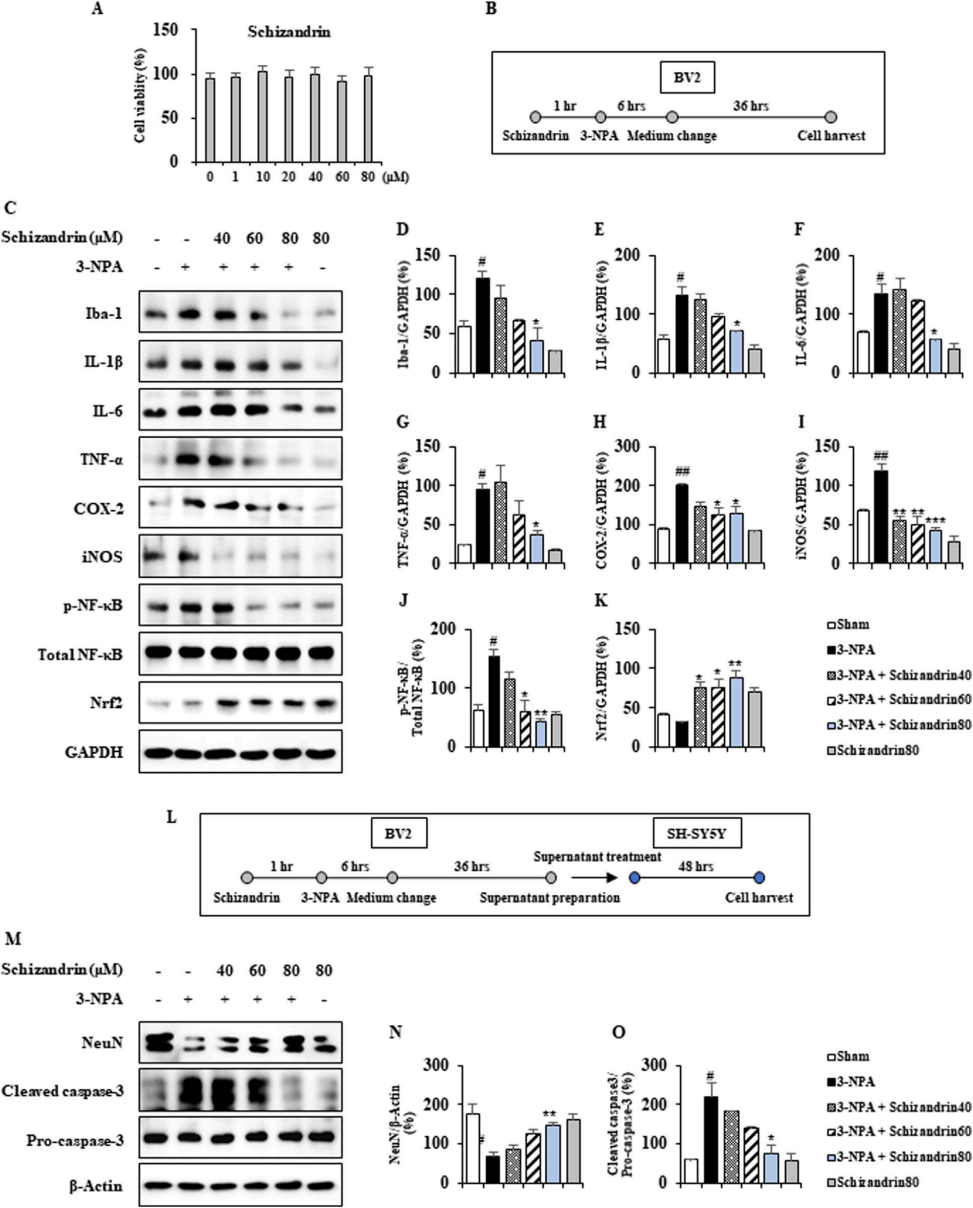
Microglial conditioned medium prevents apoptosis (neuronal cell death) in 3-NPA-induced SH-SY5Y cells. **(A)** The MTT assay was used to measure the cytotoxicity (loss of viable cells) of SOSC in BV2 cells. **(C–K)** Cultured BV2 cells were pretreated with schizandrin (40, 60, and 80 μM) 1 h before 3-NPA stimulation. These cells were subjected to Western blot analysis using antibodies against Iba-1, IL-1β, IL-6, TNF-α, COX-2, iNOS, NF-κB, and Nrf2. Their bands were quantified relative to GAPDH levels **(B–D)**. **(L–O)** Cultured SH-SY5Y cells were treated with CM-Sch-3-NPA or CM-3-NPA 1 h before 3-NPA stimulation. These cells were then subjected to Western blot analysis using NeuN and cleaved caspase-3 antibodies (E and F). Bands were quantified relative to GAPDH levels. Data are expressed as mean ± SEM (one-way ANOVA with *post hoc*; #*p* < 0.05, ##*p* < 0.01, and ###*p* < 0.001 vs. Sham group; *p < 0.05, **p < 0.01, and ***p < 0.001 vs. 3-NPA group). 3-NPA, 3-nitropropionic acid; Iba-1, ionized calcium-binding adapter molecule 1; NF-κB, nuclear factor-kappa B; Nrf2, nuclear factor erythroid 2-related factor 2; GAPDH, glyceraldehyde 3-phosphate dehydrogenase; SOSC, seed oil of *Schisandra chinensis.*

SOSC ameliorated 3-NPA-induced HD-like symptoms and neuropathology through its anti-inflammatory and antioxidant mechanisms by inhibiting microglial activation in the striatum from 3-NPA-intoxicated HD model (Figures 3–8). According to the MTT assay, the cell viabilities by Schizandrin were not significantly affected by at least 80 μM (Figure 9A). To further understand SOSC’s action mechanism, cultured BV2 cells (cell line for microglia) were stimulated with schizandrin (a main component of SOSC) (40, 60, and 80 μM) at 1 h before 3-NPA stimulation. As a result, schizandrin treatment significantly decreased protein expression levels of Iba-1 (a representative marker of microglial activation), representative proinflammatory agents (IL-1β, IL-6, TNF-α, COX-2, and iNOS), and p-NF-κB (a representative proinflammatory pathway), but significantly enhanced Nrf2 in a dose-dependent manner after 3-NPA treatment (Figures 9B–K). Subsequently, to examine whether conditioned medium from schizandrin/3-NPA-treated BV2 cells (CM-Sch-3-NPA) could reduce neurodegeneration (apoptosis) of cultured SH-SY5Y cells (a neuroblastoma cell line) compared to conditioned medium from 3-NPA-treated BV2 cells (CM-3-NPA), cells were treated with CM-Sch-3-NPA or CM-3-NPA. As a result, CM-Sch/3-NPA clearly increased protein expression levels of NeuN (a marker for neuronal cells) but and reduced cleaved caspases-3 (a marker for apoptosis), in a dose-dependent manner compared to treatment with CM-3-NPA after 3-NPA treatment (Figure 9L–O). These results suggest that schizandrin may inhibit neuronal death and apoptosis of 3-NPA-induced SH-SY5Y cells through its anti-inflammatory and antioxidant activities in 3-NPA-induced BV2 cells.

## 4 Discussion

In the present study, the administration of SOSC ameliorated movement dysfunction, improved survival rate, and inhibited neurodegeneration associated with the reduced apoptosis in the striatum following 3-NPA intoxication. These results were also consistent with reduction of microglial activation, the downregulation of pro-inflammatory mediators, and the upregulation of anti-inflammatory mediators by inhibiting MAPKs/NF-κB signaling pathways and stimulating Nrf2/HO-1 signaling pathway. Intriguingly, schizandrin, a component of SOSC reduced the protein expression levels of Iba-1 and p-NF-κB in 3-NPA-induced BV2 cells (murine microglia cell line). The conditioned medium of BV2 cells inhibited cleaved caspase-3 in 3-NPA-induced SH-SY5Y cells (a neuroblastoma cell line). In conclusion, SOSC might be a potential therapeutic agent for treating HD-like symptoms and neuropathology through its anti-inflammatory and antioxidant activities.

3-NPA, an inhibitor of succinate dehydrogenase at complex II of the mitochondrial electron transport chain induces cellular energy deficit and oxidative stress-related neurotoxicity (Brouillet et al., 2005; Huang et al., 2006; Tunez et al., 2010). It is known that 3-NPA can induce striatal degeneration through its neurotoxic activity in rodents and result in gait abnormalities, which mimicking the behavioral dysfunction and pathology caused by mutant Htt in animal models for HD and its patients (Brouillet et al., 2005; Huang et al., 2006; Tunez et al., 2010). However, the model does not involve mutant *Htt* expression (Mehrotra and Sandhir, 2014; Tunez et al., 2010). Nevertheless, the model has been steadily used to discover therapeutic interventions for HD (Kaur et al., 2023).

Herein, the amelioration of behavioral dysfunction by SOSC was closely associated with reduced levels of striatal cell death based on cresyl violet staining following 3-NPA-intoxication (Figures 3, 4). SOSC’s neuroprotective activity was also related to a decreased level of cleaved caspase-3 in the striatum (Figure 4). Staining for cleaved caspase-3 (an initiator of intrinsic apoptosis) is commonly used to label apoptotic cells in brain tissue (D'Amelio et al., 2010). These results suggest that SOSC might ameliorate movement dysfunction by inhibiting neuronal cell death via anti-apoptotic activity in the striatum following 3-NPA-intoxication.

Microglia, as brain-resident immune cells, have been identified as crucial players in regulating essential pathways in neurodegeneration and neuroinflammation (Kabba et al., 2017; Tai et al., 2007). Clinical studies using positron emission tomography have also demonstrated that microglial activation levels increase in proportion to the severity of HD symptoms Clinical studies using positron emission tomography have also demonstrated that microglial activation levels increase in proportion to the severity of HD symptoms (Pavese et al., 2006; Yang et al., 2017). Normally, 3-NPA can induce apoptosis by generating superoxide radicals (Dedeoglu et al., 2002) and then recruiting and activating microglia surrounding apoptotic cells in the striatum (Brouillet, 2014). Cell death (apoptosis) caused by the latter is called secondary cell death or delayed cell death (Giulian and Vaca, 1993). Recruited and activated microglia around or within lesions can produce pro- or anti-inflammatory mediators that are either beneficial or deleterious to neuronal survival (Lobsiger and Cleveland, 2007). Inflammatory response driven by microglia has been considered as a key contributor to the pathogenesis of several neurodegenerative diseases including HD (Rodriguez-Gomez et al., 2020). Thus, controlling microglial recrutment/activation and its pro- and anti-inflammatory mediators has been considered as an attractive anti-apoptosis (neuroprotection) strategy to protect neurons in various pathological environments (Lobsiger and Cleveland, 2007). Here, SOSC downregulated microglial activation (levels of Iba-1 immunoreactive cells and protein expression) in the striatum following 3-NPA-intoxication (Figure 5). It also downregulated mRNA levels of pro-inflammatory cytokines (IL-1β and IL-6) and enzymes (COX-2 and iNOS) but upregulated mRNA levels of anti-inflammatory cytokines (IL-10 and TGF-β) and agents (argenase-1) (Figure 6). Taken together, our findings suggest that SOSC might block striatal degeneration (apoptosis) by positively regulating pro- and anti-inflammatory responses via inhibition of microglial activation in the striatum after 3-NPA-intoxication.

NF-κB and MAPKs (JNK, ERK, and p38) signaling pathways are pivotal transcription factors for microglial activation and production of cytokines such as IL-1β, IL-6, and TNF-α in various neurodegenerative diseases (El Kasmi et al., 2006; Kwon et al., 2017). It is known that 3-NPA can cause striatal degeneration by regulating the activation of NF-κB and MAPKs signaling pathways in the striatum (Hsieh et al., 2003; Ibrahim and Abdel Rasheed, 2022; Kim et al., 2017). Here, we demonstrated for the first time that pretreatment with SOSC could inhibit the activation of both pathways in the striatum associated with improved 3-NPA-intoxication induced movement dysfunction and survival rate (Figure 3). Our findings demonstrate that SOSC may inhibit NF-κB and MAPKs activities in the 3-NPA-intoxicated striatum, in agreement with reduced inflammatory responses (Figure 7). To the best for our knowledge, little is known about the *in vivo* effect of SOSC on MAPKs and NF-κB pathways in neurodegenerative diseases including HD. Our findings firstly suggest that SOSC might be a potential HD therapeutic by inducing the downregulation of NF-κB and MAPKs pathways.

The oxidative stress is involved in the development and progression of various neurodegenerative disorders such as HD (Singh et al., 2019; Teleanu et al., 2022). Nrf2 knock-out mice show more aggravated 3-NPA- or malonate-induced motor deficits, striatal SDH activity, LDH activity, and striatal lesions, as compared to wild type mice (Tarozzi et al., 2013). Antioxidant therapies have positive effects in *in vivo* and *in vivo* model of HD (Gao et al., 2014; Gil-Mohapel et al., 2014; Suzuki et al., 2013). Oral treatment of *tert*-butylhydroquinone, an Nrf2 activator attenuates 3-NPA-induced striatal degeneration in wild type mice, but not in Nrf2 KO mice (Tarozzi et al., 2013). These reports suggest that Nrf2 is an attractive therapeutic target for HD. In this study, pretreatment of SOSC upregulated protein expression of Nrf2 and a representive phase II enzyme (HO-1) in the striatum, corresponding to changes in movement dysfunction and stritial cell death after 3-NPA-intoxication (Figure 8). We have previously reported that pretreatment with Nrf2 pathway activators (DMF and AI-3) before 3-NPA-intoxication can reduce neurological impairment and lethality (Jang and Cho, 2016). Our findings suggest that SOSC might be an essential inducible factor in the protection against 3-NPA-induiced striatal cell death, strongly suggesting its potential therapeutic role in treating HD via Nrf2-HO-1 pathway regulation.

Microglial inflammatory meduators (cytokines and enzymes) can mediate microglia-neuron interaction in neurodegenerative diseases such as HD (Hodes et al., 2015). SOSC reduced neurodegeneration and inhibited microglial activation (Figure 5). Such effects of SOSC were related to the downregulation of pro-inflammatory mediators and upregulation of anti-inflammatory mediators (Figure 6). Therefore, we hypothesized that microglial downregulation by SOSC might affect neuronal apoptosis via secreted cytokines, resulting in striatal degeneration and behavioral dysfunction. As a result of testing this hypothesis, SOSC and its a main component schizandrin inhibited mRNA or protein expression levels of representative pro-inflammatory cytokines (IL-1β, IL-6, or TNF-α), enzyme (COX-2 and iNOS), and p-NF-κB in not only 3-NPA-intoxicated striatum, but also in 3-NPA-stimulated BV2 cells (Figures 5–7, 9). More importantly, CM-Sch/3-NPA clearly reduced protein expression level of cleaved caspases-3 (a marker for apoptosis) in 3-NPA-induced SH-SY5Y cells (a neuroblastoma cell line) in a dose-dependent manner (Figure 9E). Schizandrin A/C can inhibit microglia-mediated neuroninflammation by inhibiting NF-κB and Nrf-2 signaling pathways (Park et al., 2013; Song et al., 2016). It can also inhibit neuroinflammation by reducing pro-inflammatory cytokines through regulation of NF-κB/NLRP3/Iba-1 signaling (Yan et al., 2021). Similarly, gomisin A significantly reduced mRNA expression and production of pro-inflammatory factors, including TNF-α, IL-1β, and IL-6, in LPS-stimulated N9 microglial cells without causing cytotoxicity. It also inhibited COX-2 and iNOS expression, suppressed ROS production, NADPH oxidase activation, and gp91phox expression, and blocked NF-κB and MAPK signaling pathways. Additionally, it alleviated cell death in SH-SY5Y neuroblastoma cells induced by conditioned media from activated microglia. Gomisin N also showed anti-inflammatory effects by inhibiting inflammation-related gene expression in LPS-stimulated BV-2 cells. *In vivo*, gomisin N reduced LPS-induced gene expression and c-Fos-positive cells in the hypothalamus and amygdala. It also alleviated depressive-like behaviors in the forced swim test and improved exploratory deficits in LPS-treated mice. Taken together, our findings suggest that SOSC including schizandrin, gomisin A, gomisin N might inhibit neurodegeneration (apoptosis) through its anti-inflammatory and antioxidant activities via inhibition of microglial activation in the striatum after 3-NPA-intoxication.

## 5 Conclusion

**FIGURE 10:**
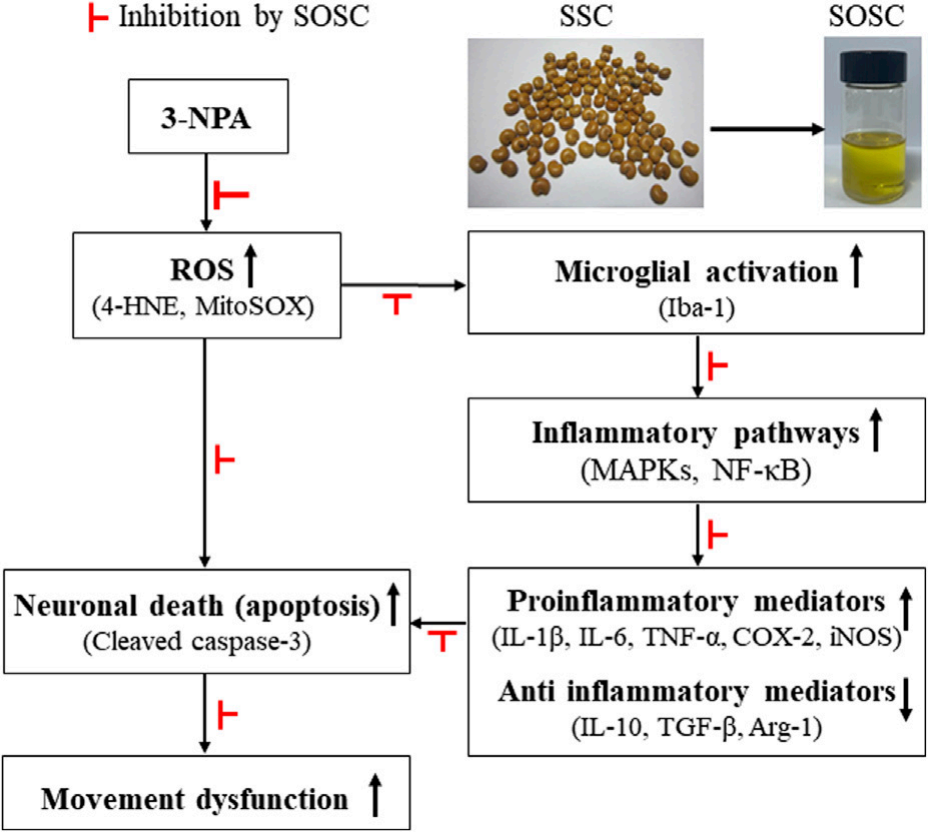
A supercritical oil extract of *Schisandra chinensis* seeds ameliorates Huntington’s disease-like symptoms and neuropathology: The potential role of antioxidant and anti-inflammatory effects. 3-NPA, 3-nitropropionic acid; 4-HNE, 4-hydroxynonenal; ARG1, arginase-1; Iba-1, ionized calcium-binding adapter molecule 1; COX-2, cyclooxygenase-2; IL, interleukin; iNOS, inducible nitric oxide synthase; MAPKs, mitogen-activated protein kinases; MitoSOX, mitochondrial superoxide indicator; NF-κB, nuclear factor-kappa B; Nrf2, nuclear factor erythroid 2-related factor 2; ROS, reactive oxygen species; TGF-β, transforming growth factor-β; TNF-α, tumor necrosis factor-alpha; SOSC, seed oil of *Schisandra chinensis;* SSC, seed of *Schisandra chinensis*.

Valuable therapeutics for HD-like symptoms have not been fully elucidated yet. Here, we demonstrate that SOSC could ameliorate striatal degeneration and neuropathology associated with a reduced inflammatory response and enhance anti-inflammatory and antioxidant events by inhibiting MAPKs and NF-κB signaling and stimulating Nrf2 signaling in the striatum (Figure 10). Despite the relative lack of information on the efficacy and mechanisms of action of SOSC, our findings strongly suggest that SOSC might be used as a potential therapeutic to ameliorate HD-like symptoms and neuropathology by regulating inflammatory and antioxidant pathways. Our results also suggest that it is necessary to determine the efficacy and mechanisms of action of SOSC in various pathological conditions including neurological disease in the future and to identify its chemical interactions *in vivo*.

## Data Availability

The datasets presented in this study can be found in online repositories. The names of the repository/repositories and accession number(s) can be found in the article/supplementary material.
